# Michael Scheeringa

**DOI:** 10.1192/bjb.2023.84

**Published:** 2024-02

**Authors:** Abdi Sanati

**Figure:**
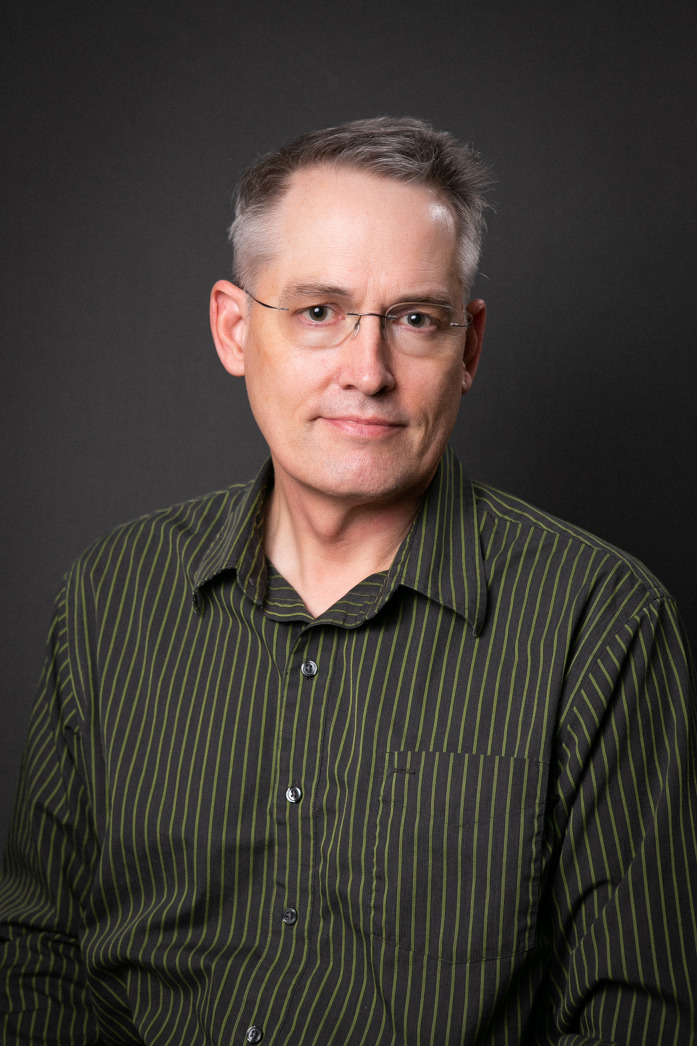

In recent years, we have witnessed the rise of trauma and trauma-related concepts in psychiatry. I have been fascinated by how quickly trauma became the new master narrative, and as a colleague pointed out, psychiatry should be aware of such master narratives. I always believed there is a need for an *advocatus diaboli* to counter-argue against any dominant ideology and my search led to the work of Professor Michael Scheeringa. Professor Scheeringa retired last year from Tulane University School of Medicine, New Orleans. He has almost three decades of clinical and research experience in field of post-traumatic stress disorder (PTSD) in children. His book *The Trouble with Trauma* was published in 2022, which I enjoyed reading. We had not been acquainted, nevertheless we had an interesting conversation, part of which you can read in this interview.

**Professor Scheeringa, many thanks for agreeing to this interview. In your opinion, why has trauma become so dominant in English-speaking psychiatry? I don't know whether in continental Europe or Japan it's that big, but I see in the English-speaking world trauma is very dominant. Why do you think that has happened?**

I think it's ideology and personal politics. The whole world is fracturing right now along liberal and conservative ideologies and trauma is fast becoming one of the institutional orthodoxies that fits right along with a lot of other liberal projects. So we're getting right into politics with this, but that's what this is all about. At the end of the day, it's not about scientific evidence. It's an ideology saying we need to expand the definition of trauma. So, to be more specific, as I have mentioned in my book, very humanitarian people like paediatricians and psychiatrists wanted to do things to help stop violence and stop child abuse. We have done a lot of things over the last century that made dents in that. But then we hit a wall and they haven't reached this utopia of preventing violence. So, they decided they needed a way to scream louder. Just like a lot of extreme causes, whether it's saving the whales, or animal rights or other political issues, you need to get attention and they realised trauma was the way to get attention and they invented this idea of toxic stress. Toxic stress asserts that psychological stress can permanently damage the brain. And once they figured that out, and it started working, they were off to the races and we've had 20 years or more now of this expansion of trauma that's based on an ideology and then they searched for the facts that fit their belief.

**It is interesting because the definition of trauma has become much wider and I was thinking that it has been stretched so thin that it covers almost everything. I remember a colleague once told me that everything is trauma, and that reminded me of Karl Popper, who said that the theory that explains everything explains nothing. So in a way, my fear is actually we end up not explaining anything with trauma because it's becomes ubiquitous.**

Right, it has become a weapon that can explain anything. That has exactly been the strategy. You've got trauma expanded to include stress, things that are not life-threatening issues. You've got the adverse childhood experiences – or ACEs – literature that says trauma can cause this array of physical illnesses. So it is literally this platform that explains how any experience of life can mould a human being and activists can use that to fit whatever political, social agenda they have.

**I am amazed at how fast it has become so big. Many years ago when I taught medical students, I used an analogy that there's a tripod and if any condition satisfies all three legs it will be sanctioned easily, the legs being the academics, the profiteers and the activists. It seems that they all agreed very quickly on trauma and it has become so big.**

Yes and I'd say two things about that. One, I think that the way it spread so fast is a reflection of our field. It is documented in research that mental health practitioners are self-identified liberals, like over 90% of our field. Jordan Peterson has said he may be the only conservative psychologist he knows. And that's an exaggeration, but you get the point. Academia is the same way. They've done studies, and academics, particularly in psychology and psychiatry, are over 90% self-identified as liberal. So, that has made this spread so easily with so few conservatives who can speak out against it.

Second, these efforts to promote trauma hype bypassed the democratic process. In a democracy, the usual process of generating agreement is to educate the citizens, have debate and develop a consensus among informed citizens. But because of the dominance in our field of like-minded believers, the agreement on wrong trauma beliefs has been so quick because it skipped this lengthy democratic process. For example, I documented in my book how the phrase ‘toxic stress’ was invented by an activist group at Harvard as a strategy to get attention. Also, I've had my blogs censored, peer review comments censored and papers rejected because I wrote about facts, backed up by the evidence, that disagreed with the consensus beliefs about trauma hype. And there were no avenues of appeal, which pretty much describes an authoritarian system in our professional organisations.

**You have spoken in your book about the scientific basis of trauma. Is there much proper scientific basis to it?**

Well, there is, in terms of whether the definition of trauma should be expanded beyond life-threatening events. There's a very good scientific basis that says trauma is really only life-threatening experiences, that are sudden and unexpected, that cause post-traumatic stress disorder. And you can point to some of the studies on non-life-threatening things like neglect or emotional abuse or watching violence on television, or a child with divorced parents, which claimed that those people may have elevated rates of PTSD. But I think those are almost all false positives because they are based on bad methodology of self-report checklists. They are not based on interviews. They cannot really understand if a symptom is truly a symptom that involves functional impairment. There are tonnes of studies like this that people can cite that I think are all bad. So in that sense, I think research is really solid. It's only life-threatening events that cause trauma. But there is the other component of things like toxic stress, which is the notion that psychological stress can permanently damage the brain. Again, there's good research and there's bad research. The proponents of that theory always cite cross-sectional research. Everybody knows correlation is not causation. You can't make a causal conclusion about events from a cross-sectional study. There are now about 28 prospective studies where people have been studied prior to trauma experiences and then followed after they've had trauma experiences. Usually these are done with soldiers, policemen and so forth. I have written a review paper which described all these studies, and the pre-trauma prospective studies do not support toxic stress. They show that any brain differences were present prior to trauma experiences. And that's why you see the associations.

**A question that came to me was could we really generate that level of evidence? Given the fact that the effect sizes are not that big we need huge samples that we follow for a long time. Could anybody actually generate that? The funding challenge for that kind of research could be enormous.**

That would be a lot of work but if they're serious about promoting this belief in toxic stress, and trying to find evidence for it, then what they have to do is to be serious about the research and follow people before their traumatic experiences and follow them over a long time. Yes, those are expensive studies, but even at that, I think they would be premature. Whenever there's a new disorder in medicine you start with case studies. It means you first have to have one person that has this new so-called disorder with a solid case report about them or just a handful of case reports. There's not a single case report in the world of a person who was studied and who had zero brain abnormalities before trauma, then experienced trauma and then developed these brain differences. If you are just doing case reports, it is not expensive. It will take time. There is not even a case study of toxic stress being true.

**The question for me is even when we have some changes in neuroimaging studies, would those changes translate to the claims that they make about symptomatology and the long-term effects of trauma?**

Well, that's a great point. Even if you find brain differences, it has to translate into functional impairment and all these serious deficits that people supposedly develop. The brain is a lot more complicated and a lot more adaptive than the promoters of trauma think. Maybe trauma changes some neurons and some functional activity in the brain. But the brain is amazingly adaptive and can handle changes.

**I remember this article about a man who had a headache and he had a scan. The doctors found he had a massive hydrocephalus and his brain was compressed to one inch underlying the skull, and he was functioning, living, having a family, paying his taxes, etc. And it was surprising how adaptive our brains are. To say on the basis of some small changes on fMRI that this person is disordered is always beyond me. In your book, you actually refer to trauma and its comparison with genetics. I recently did an interview with a colleague who is a professor of genetics, and this question was raised there too. Do you think genetics is being ignored for things like trauma or social determinants, which are very much in vogue?**

Yes, because if they accept the premise that genetics is the cause of brain differences or people being disadvantaged, that is directly contrary to their ideology that nurture causes all the problems. I am not sure how far you want to get into this, but to say that nurture and life experiences are what causes all of people's problems in the world, it is a cultural Marxism theory. A theory that says outside forces oppress people and mould them, therefore we need a revolution against all the outside forces. That is basically kind of what the toxic stress theory is saying: that all people's problems are caused by trauma. So we need a revolution in society, to overthrow all these forces.

**That takes us back to the politics, because it's kind of a political issue. Currently in the Western world, as you also have mentioned, the dominant politics is the politics of victimhood and trauma fits very well into that.**

It is an ideology of victimhood. It gives you an endless array of things to fight against. When people have this certain type of politics that we're talking about, as you know, kind of progressive liberal, politics is what gives them meaning in their lives and having things to fight for is what gives them meaning. So they need endless fighting. They need endless things to fight against. And that's what trauma gives them – a perfect platform for endless fighting.

**One concept that is discussed a lot here is intergenerational trauma; now we are talking about reparations for the past, and the sins of the fathers. I think intergenerational trauma fits well in that context, too. Because how am I to go to someone and say, because your ancestors did harm to my ancestors, you have to pay me reparations. He hasn't done anything to me, but if I say that, I'm saying I have suffered intergenerational trauma because of the trauma that happened to my ancestors. So it gives me a claim to something.**

Intergenerational trauma is another one of these things that people have accepted and there is really no evidence for it. Maybe there are some rat studies that show you that how a mother licks the pups can influence them. But humans are much more complex than rats and humans are not rats. And for intergenerational transmission of trauma to work, you have to have a mechanism for how that happens. This used to be called Lamarckian genetics that, as you know, if a giraffe stretches its neck to reach leaves up in a tree then its offspring is born with a longer neck. The way that people now think that can happen is a kind of a neo-Lamarckian genetics through epigenetics, DNA methylation. And there is just no evidence that a fetus can get its DNA methylated in the womb to pass on acquired characteristics of parents. Passing on DNA just doesn't work that way.

**One other thing I really liked about your book was when you spoke about the abandonment of truth and that is, again, something political, especially with the rise of postmodern politics.**

That is the central issue. I mean, a researcher or a clinician is supposed to be an expert in the world on a certain topic, and they are supposed to know what the truth is. We are experts as researchers or clinicians. We are the people in the tribe that other people come to for the truth. And if we do not stick to the truth what is the point of us having any role of expertise? If we just become people who espouse our belief systems in our ideology, because we think that's the way we want the world to work, we are no longer experts who can be relied on.

**There is another political aspect of taking truth out of expertise, so everybody could identify as a kind of an expert. That brings in the politics of lived experience that people talk about their truth.**

Right, that's the common retort, like, I have lived experience that you don't have. Well, that is not evidence. That is an anecdote. An anecdote is not usually what we accept as scientific evidence; that bar is a little bit higher than anecdotes and lived experience.

**You mentioned trauma and social engineering and activism. Could you tell us a bit more about that for the readers in the UK who are not familiar with your work?**

The main thing I would want people to understand about all of this is the expansion of trauma, all the trauma hype. We get caught up in talking about the evidence and the things that people are doing, but at the end of the day, you really have to talk about the people who are doing it and their personal belief systems. It really is their motivation, their meaning in life. The reason they get up every day and find meaning in their life is to fight for social re-engineering. That's their motivation. And that's why these movements have been so strong and people cling to them so fiercely, because challenging trauma hype challenges their very basic meaning in life.

**In your book, you compared the rise of trauma with the rise of false memories or the schizophrenogenic mother. Do you think that trauma is going to go the same way as those concepts have gone?**

I think we are going to reach a stalemate. I think there just are people in the world who will always want to believe the trauma hype. That will always be their comfort zone. We are not going to be able to reason it out of them. And right now we just need to balance the discussion. The discussion is very one-sided in favour of this trauma hype and by speaking out in venues like this we need to bring some equipoise to the discussion that has never been there. And if we can just have at least a stalemate, where the discussion is balanced, then people can make up their own mind without being swamped with dogma and being indoctrinated in all the so-called training programmes. That is my big hope. At least in my lifetime.

